# Publisher Correction: Pluripotent stem cell-derived model of the post-implantation human embryo

**DOI:** 10.1038/s41586-023-06524-4

**Published:** 2023-08-17

**Authors:** Bailey A. T. Weatherbee, Carlos W. Gantner, Lisa K. Iwamoto-Stohl, Riza M. Daza, Nobuhiko Hamazaki, Jay Shendure, Magdalena Zernicka-Goetz

**Affiliations:** 1 Department of Physiology, Development and Neuroscience, University of Cambridge, Cambridge, UK; 2 Department of Genome Sciences, University of Washington School of Medicine, Seattle, WA USA; 3 Brotman Baty Institute for Precision Medicine, Seattle, WA USA; 4 Howard Hughes Medical Institute, Seattle, WA USA; 5 Allen Discovery Center for Cell Lineage Tracing, Seattle, WA USA; 6 Division of Biology and Biological Engineering, California Institute of Technology, Pasadena, CA USA

**Keywords:** Stem cells, Embryogenesis, Stem-cell differentiation

Correction to: *Nature* 10.1038/s41586-023-06368-y Published online 27 June 2023

In the version of this article initially published, ref. 18 (Simunovic, M. et al., *Cell Stem Cell*. 10.1016/j.stem.2022.05.001 (2022)) was missing, and in Fig. 1a,b, yellow header labels for “GATA6” and “GATA3” were missing. In addition, in the Methods section “Generation of inducible human ES cell lines”, in the text now reading “… pBase plasmid expressing PiggyBac Transposase using the Neon transfection system with the following settings: 1,200 V, 20 ms, 2 pulses”, 20 ms first appeared in error as “2 ms”. The corrections have been made in the HTML and PDF versions of the article.

