# Supplementary material for: Pluripotent stem cell-derived model of the post-implantation human embryo
Source: Nature. 2023 Jun 27;622(7983):584–93. doi: 10.1038/s41586-023-06368-y (PMC10584688; doi:10.1038/s41586-023-06368-y)
Supplement: Supplementary file 1 — Reporting Summary [file 41586_2023_6368_MOESM1_ESM.pdf]

nature portfolio

Corresponding author(s): Magdalena Zernicka-Goetz

Last updated by author(s): Jun 14, 2023

Reporting Summary

Nature Portfolio wishes to improve the reproducibility of the work that we publish. This form provides structure for consistency and transparency in reporting. For further information on Nature Portfolio policies, see our Editorial Policies and the Editorial Policy Checklist.

Statistics

For all statistical analyses, confirm that the following items are present in the figure legend, table legend, main text, or Methods section.

n/a	Confirmed	
☐	☒	The exact sample size (*n*) for each experimental group/condition, given as a discrete number and unit of measurement
☐	☒	A statement on whether measurements were taken from distinct samples or whether the same sample was measured repeatedly
☐	☒	The statistical test(s) used AND whether they are one- or two-sided<br>*Only common tests should be described solely by name; describe more complex techniques in the Methods section.*
☒	☐	A description of all covariates tested
☐	☒	A description of any assumptions or corrections, such as tests of normality and adjustment for multiple comparisons
☐	☒	A full description of the statistical parameters including central tendency (e.g. means) or other basic estimates (e.g. regression coefficient) AND variation (e.g. standard deviation) or associated estimates of uncertainty (e.g. confidence intervals)
☐	☒	For null hypothesis testing, the test statistic (e.g. *F*, *t*, *r*) with confidence intervals, effect sizes, degrees of freedom and *P* value noted<br>*Give P values as exact values whenever suitable.*
☒	☐	For Bayesian analysis, information on the choice of priors and Markov chain Monte Carlo settings
☒	☐	For hierarchical and complex designs, identification of the appropriate level for tests and full reporting of outcomes
☒	☐	Estimates of effect sizes (e.g. Cohen's *d*, Pearson's *r*), indicating how they were calculated

Our web collection on statistics for biologists contains articles on many of the points above.

Software and code

Policy information about availability of computer code

Data collection	No software was used for Data Collection.
Data analysis	No custom code was used in this manuscript. Scripts to used to analyze these data is available at:<br>https://github.com/bweatherbee/human_model<br>List of software used -<br>command line tools: CellRangerARC (2.0.0)<br>R - R (4.1.2); Seurat (4.2.0), Signac (1.8.0), EnsDb.Hsapiens.v86 (2.99.0), dplyr (1.0.10), ggplot2 (3.3.6), Scillus (0.5.0), RColorBrewer(1.1-3), magrittr (2.0.3), cowplot(1.1.1), viridis (0.6.2), data.table (1.14.2), scmap (1.16.0), biomaRt (2.50.3), SingleCellExperiment (1.16.0), tidyverse (1.3.2), scDblFinder (1.11.4), SCpubr (1.1.2), switchde (1.3.2), scillus (0.5.0)<br>Python - CellPhoneDB (2.1.7), python (3.8), macs2 (2.2.7), scvelo (0.2.3), matplotlib (3.3.0), anndata (0.7.5), scanpy (1.5.1), numpy(1.17.5), multivelo (0.1.2)<br>GraphPad Prism (9.4), FIJI (2.1.0), Imaris (9.1.2)

For manuscripts utilizing custom algorithms or software that are central to the research but not yet described in published literature, software must be made available to editors and reviewers. We strongly encourage code deposition in a community repository (e.g. GitHub). See the Nature Portfolio guidelines for submitting code & software for further information.

Data

Policy information about availability of data

All manuscripts must include a data availability statement. This statement should provide the following information, where applicable:

- Accession codes, unique identifiers, or web links for publicly available datasets
- A description of any restrictions on data availability
- For clinical datasets or third party data, please ensure that the statement adheres to our policy

For aligning sequencing data, GRCh38 (https://www.ncbi.nlm.nih.gov/assembly/GCF_000001405.26/) and GRCm38 (https://www.ncbi.nlm.nih.gov/assembly/GCF_000001635.20/) were used.

Previously published data is publicly available:
Human data
Molè et al., 2021: ArrayExpress E-MTAB-8060
Xiang et al., 2020: Gene Expression Omnibus GSE136447
Zhou et al., 2019: Gene Expression Omnibus GSE109555
Petropoulos et al., 2016: ArrayExpress E-MTAB-3929
Blakely et al., 2015: Gene Expression Omnibus GSE66507
Yan et al., 2013: Gene Expression Omnibus GSE36552

Cynomolgus Monkey
Yang et al., 2021: Gene Expression Omnibus GSE148683
Ma et al., 2019: Gene Expression Omnibus GSE130114
Nakamura et al., 2016: Gene Expression Omnibus GSE74767

hESC derived datasets
Pham et al., 2022: Gene Expression Omnibus GSE191286
Kagawa et al., 2022: Gene Expression Omnibus GSE177689
Zheng et al., 2019: Gene Expression Omnibus GSE134571

10x multiome data for cell lines and inducible human embryoids: Gene Expression Omnibus GSE218314

Source Data are provided with this manuscript

Human research participants

Policy information about studies involving human research participants and Sex and Gender in Research.

Reporting on sex and gender	We do not have access to prenatal genetic testing for the vast majority of embryos. Therefore, the composition of sex chromosomes of embryos cultured in the lab is largely unknown.
Population characteristics	According to the United Kingdom's Human Fertilisation and Embryology Act, which governs human embryo research, identifiable information of parents donating embryos to research is redacted. Therefore population characteristics of donating patients and their embryos is unknown.
Recruitment	Human embryos are donated by patients in the UK from collaborating IVF clinics under HFEA licence R0193. Patients undergoing IVF at CARE Fertility, Bourn Hall Fertility Clinic, Herts & Essex Fertility Clinic, and King’s Fertility was given the option of continued storage, disposal, or donation of embryos to research (including project specific information) or training at the end of their treatment. Patients were offered counseling, received no financial benefit, and could withdraw their participation at any time until the embryo had been used for research.<br><br>All information of patients is required to be redacted prior to donation to research. Therefore, potential biases based on recruitment is unknown. Please note this manuscript does not perform any experimentation on human embryos, rather we seek to provide a single example of an embryo cultured in vitro as a reference image for the natural post-implantation embryo.
Ethics oversight	Ethical oversight is provided both by the HFEA and the Human Biological Research Ethics Committee at the University of Cambridge. The recruitment of patients to donate human embryos to research follows the Human Fertilisation and Embryology Authority's guidelines. This includes the provision of project-specific information, the offering of counseling, and the ability to withdraw consent at any time until the embryos have been used. Stem cell work is approved by the UK Stem Cell Bank.

Note that full information on the approval of the study protocol must also be provided in the manuscript.

Field-specific reporting

Please select the one below that is the best fit for your research. If you are not sure, read the appropriate sections before making your selection.

☒ Life sciences ☐ Behavioural & social sciences ☐ Ecological, evolutionary & environmental sciences

For a reference copy of the document with all sections, see nature.com/documents/nr-reporting-summary-flat.pdf

Life sciences study design

All studies must disclose on these points even when the disclosure is negative.

Sample size	No tests were used to predetermine sample size. Sample sizes for experimentation was determined based on our previous experience with human embryos (Shahbazi et al., 2016, Mole et al., 2021), human stem cells and 3D stem cell models (Shahbazi et al., 2017, Mackinlay et al., 2021), and mouse embryoid models (Harrison et al., 2017, Sozen et al., 2018, Amadei et al., 2021, Amadei et al., 2022, Lau et al., 2022).<br><br>Embryoid generation depends on the aggregation of stem cells in Aggrewell dishes which contain 1200 individual microwells. Individual structures are then recovered for analysis. Given intra-experiment variability, it is difficult to predict the number of individual samples each experiment will yield. Therefore, rather than determining predetermined sample sizes, we ensured all experiments were reproduced between 2 researchers across multiple independent experiments and cell lines.
Data exclusions	Exclusion criteria for single cell sequencing data was as follows to ensure high quality barcodes were used: Cells with >500 RNA UMI counts, <20% mitochondrial reads, >500 ATAC reads, TSS enrichment >1 and were called as singlets using scDblFinder were retained for downstream analysis.<br>For human embryoid efficiency quantifications and quantifications of cell line qPCR or immunofluorescence data, no data was excluded.<br>For quantification of 'inner domain' expression patterns or anterior hypoblast expression patterns of human embryoids (Figures 4 and 5), as well as selection of structures to subject to single cell sequencing, only aggregates with an organized epithelial inner domain, an intermediate tissue, and an outer layer of GFP-positive cells were included as these are the criteria that we define the human model by.
Replication	All experiments were repeated independently across multiple freeze-thaw cycles, and multiple conversions to the naive pluripotency state. Generation of inducible human embryoids was also repeated in 2 hESC backgrounds (Shef6 and RUES2). All experiments were performed independently at least twice. Embryoid generation was performed over 100 independent experiments throughout the duration of this project. Embryoid experimentation was performed by 2 authors and results reproduced consistently.
Randomization	Randomization is not relevant to this study. In experiments were embryoids were allocated to different groups (i.e. the addition of small molecule inhibitors), the media containing small molecules were added directly into Aggrewells where embryoids were generated. Given that embryoids were generated and treated within Aggrewell dishes, which contain 1200 microwells where individual embryoids may develop, and treatment between days 0-2 or days 2-4 was performed within these dishes, it would not have been possible to randomly allocate individual structures as they were developing within the dish.
Blinding	Investigators were not blinded to experimental groups. Given the necessity to keep clones and transgenic hESC lines pure and seperated, as well as the necessary step of calculating initial plating density, it would not be possible to blind the cell populations used to generate inducible human embryoids. Additionally, it would not be feasible to blind the media changes with the addition of small molecules as we made media in-house.

Reporting for specific materials, systems and methods

We require information from authors about some types of materials, experimental systems and methods used in many studies. Here, indicate whether each material, system or method listed is relevant to your study. If you are not sure if a list item applies to your research, read the appropriate section before selecting a response.

Materials & experimental systems

n/a	Involved in the study	
☐	☒	Antibodies
☐	☒	Eukaryotic cell lines
☒	☐	Palaeontology and archaeology
☐	☒	Animals and other organisms
☒	☐	Clinical data
☒	☐	Dual use research of concern

Methods

n/a	Involved in the study	
☒	☐	ChIP-seq
☒	☐	Flow cytometry
☒	☐	MRI-based neuroimaging

Antibodies

Antibodies used	AP2-alpha (Santa Cruz Biotechnology; sc-12726; clone 3B5; 1:200); AP2-gamma (R&D Systems; AF5059; 1:500); AP2-gamma (Santa Cruz Biotechnology, sc-12762; clone 6E4/4; 1:200); Brachyury (R&D Systems; AF2085; 1:500); CDX2 (BioGenex; MU392-UC; clone

CDX2-88; 1:200); CER1 (R&D Systems; AF1075; 1:500); Cytokeratin 7 (Aligent; M7018; clone OV-TL 12/30; 1:100); E-Cadherin (BD Biosciences; 610182; clone 36; 1:200); EOMES (Abcam; ab23345; 1:200); FOXA2 (R&D Systems; AF2400; 1:200); GATA2 (Novus Biologics; NBMP1-82581; 1:200); GATA3 (Abcam; ab199428; clone EPR16651; 1:500); GATA4 (Santa Cruz Biotechnology; sc-25310; clone G-4; 1:200); GATA4 (ThermoFisher Scientific; 14-9980-82; clone eBioEvan; 1:500); GATA6 (R&D Systems; AF1700; 1:500); GATA6 (Cell Signaling Technology; 5851; clone D61E4; 1:2000); GFP (Abcam; ab13970; 1:1000); GFP (Nacalai USA; GF090R; clone GF090R; 1:1000); HAND1 (DSHB; PCRP-HAND1-2A9; clone 2A9 1:200); HNF4-alpha (Abcam; ab201460; clone EPR16885-99; 1:2000); ISL1 (DSHB; PCRP-ISL1-1A9; clone 1A9; 1:100); Laminin (Sigma Aldrich; L9393; 1:200); N-Cadherin (Abcam; ab98952; clone 5D5; 1:200); NANOG (Cell Signaling Technology; 4903; clone D73G4; 1:200); OCT3/4 (Santa Cruz Biotechnology; sc-5279; clone C-10; 1:100); OTX2 (R&D Systems; AF1979; 1:1000); phospho-SMAD1/5 (Cell Signaling Technology; 9516S; clone 41D10; 1:200); Smad2/3 (Cell Signaling Technology; 8685S; clone D7G7; 1:200); SOX17 (R&D Systems; AF1924; 1:500); SOX2 (ThermoFisher Scientific; 14-9811-82; clone Btjce; 1:500); TBX20 (R&D Systems; MAB8124; clone 668710; 1:100); VTCN1 (Abcam; ab209242; clone EPR20236; 1:200).

AlexaFluor-405 Donkey Anti-Mouse (ThermoFisher Scientific; A48257; 1:500); AlexaFluor-488 Donkey Anti-Rat (ThermoFisher Scientific; A-21208; 1:500); AlexFluor-488 Donkey Anti-Mouse (ThermoFisher Scientific; A-21202; 1:500); AlexaFluor-488 Donkey Anti-Goat (ThermoFisher Scientific; A-11055; 1:500); AlexaFluor-568 Donkey Anti-Rabbit (ThermoFisher Scientific; A10042; 1:500); AlexaFluor-568 Donkey Anti-Rat (ThermoFisher Scientific; A78946; 1:500); AlexaFluor-568 Donkey Anti-Mouse (ThermoFisher Scientific; A10037; 1:500); AlexaFluor-647 Donkey Anti-Rat (ThermoFisher Scientific; A78947; 1:500); AlexaFluor-647 Donkey Anti-Goat (ThermoFisher Scientific; A-21447; 1:500); AlexaFluor-647 Donkey Anti-Rabbit (Thermofisher Scientific; A-31573; 1:500); AlexaFluor-647 Donkey Anti-Mouse (ThermoFisher Scientific; A32787; 1:500).

Antibody table is provided in Supplementary Table 5

Validation	All primary antibodies are validated for detection of the human antigen of interest according to manufacturer's websites. Details of the validation statement, antibody profiles and relevant citations can be found on the manufacturer's website. In addition to that, all antibodies in this study showed expected staining patterns based on protein type (e.g. transcription factors in the nucleus, membrane-bound proteins at the membrane) in human embryonic stem cells.

Eukaryotic cell lines

Policy information about cell lines and Sex and Gender in Research

Cell line source(s)	UK Stem Cell Bank
Authentication	Cell lines were authenticated by STR analysis.
Mycoplasma contamination	All cell lines were regularly tested for mycoplasma contamination, and were negative.
Commonly misidentified lines (See ICLAC register)	N/A

Animals and other research organisms

Policy information about studies involving animals; ARRIVE guidelines recommended for reporting animal research, and Sex and Gender in Research

Laboratory animals	CD1 and F1 wildtype males aged 6 to 45 weeks and CD1 and F1 wildtype females aged 6 to 18 weeks were used for this study.
Wild animals	No wild animals were used in this study.
Reporting on sex	Sex was not considered in this study as embryos were recovered from the mother and used for experimentation at the 8-cell stage. Genotyping was not performed.
Field-collected samples	No field-collected samples were used in this study.
Ethics oversight	Mice were kept in an animal house on 12:12 hour light-dark cycle with ad libitum access to food and water. Experiments with mice are regulated by the Animals (Scientific Procedures) Act 1986 Amendment Regulations 2012 and conducted following ethical review by the University of Cambridge Animal Welfare and Ethical Review Body (AWERB). Experiments were approved by the Home Office under Licenses 70/8864 and PP3370287. CD1 and F1 wildtype males aged 6 to 45 weeks and CD1 and F1 wildtype females aged 6 to 18 weeks were used for this study. Animals were inspected daily and those showing health concerns were culled by cervical dislocation.

Note that full information on the approval of the study protocol must also be provided in the manuscript.
